# Monthly Memoranda

**Published:** 1853-09

**Authors:** 


### EDITORIAL DEPARTMENT.

We have met with an inconvenience, both in our connection with this and other Medical Journals. As we take a seat, here by our table, and scan this ponderous pile of exchanges, which brings its monthly freight of medical wealth to our sanctum, we feel an inward sense of regret, that we cannot transfer to our pages the numerous articles of real practical utility, which the medical literature of the country is constantly producing. We endeavor, indeed, to include, in our Eclectic Department, as many articles of practical interest as our space will admit. But, our journal, like many very excellent members of the community, is thin; it has yet hardly attained the age which brings with it a comforting corpulence. But, although the kindness of its friends warrants the promise of its enlargement at the early age of one year, yet we now feel the constraint of a limited area. We wish that we could convey to every one of our numerous patrons, a special invitation to occupy the easy chair, yonder, and enjoy the pleasant pastime of perusing the numerous articles of interest with which every mail would enable us to furnish him. But, as we cannot do this, nor find room to print at length all we would gladly select, we propose another course. We design to set apart a portion of our Editorial Department for the purpose of making a brief and concise digest, every month, of the more prominent subjects of passing interest. In this place we shall condense many articles which we cannot copy at length, in the Eclectic Department; give an analysis of many subjects which demand notice, but will not admit of full expansion, and shall endeavor to adapt it particularly to the constant wants of the practitioner. In fine, we hope to afford, hereby, a bird's eye view of the condition and improvements of medical science. We shall endeavor to carry out the objects represented, under the title of

### Monthly Memoranda.

*The Medical properties of ox gall* are attracting the attention of medical men. It is believed to be a very valuable agent in many disordered functional conditions; it is most natural to suppose that it would be of service in many cases of disease, arising from disordered or suppressed biliary secretion. The gall is evaporated in an oven, or in the sun, to a suitable firmness, and then made into pills with magnesia, pulv. glychyr, or carb. iron. A pill of about gr. v. is administered every two hours, or thrice daily, or less often, *p. r. n.* It is alternative, laxative and somewhat tonic. It acts as a natural stimulus to the alimentary canal, supplying in a degree the place of the normal secretion. In obstinate constipation, it is signally useful. Hardened, clay-colored fæces are softened, and natural healthy evacuations ensue. Many cases are reported, where long standing and extremely painful constipation has been entirely remedied by this means. In hepatic derangements of various kinds, congestion, jaundice, dyspepsia and sick headache, it has often been used with very marked success, and its employment often obviates the necessity of harsher remedies. In fevers of various grades, nervous, continued, and typhoid, its use takes the place of strong cathartics, as it has a natural and favorable laxative action. It is not an unpleasant remedy, as above prepared, and may be favorably employed in the various summer complaints of children. We earnestly recommend its more general employment.

*Scarlatina.*—Dr. Hester, of Reading, Penn., mentions the treatment of seventy-two cases of scarlatina, which he conducted during the last year. They were all treated by laxatives, when required, which was usually at the commencement, and the unrestrained use of hydrochloric acid, diluted with water, and sweetened, except in one case, where bronchitis demanded antimonials. Fifteen or twenty drops of the acid were put into half a pint of water, well sweetened with white sugar, and allowed *ad libitum.* It was a pleasant drink to the patient. No other general treatment was resorted to. Dr. H. attributes considerable efficiency to its use, yet not as a specific to be relied on, without other means. He thinks that, when early used, it has a salutary effect on the local inflammation of the throat. Externally, to the throat, he uses counter-irritants, preceded by leeching, in severe cases. A liniment of ol. tereb and ol. oliv, was applied every three or four hours; and the sloughy ulcers of the throat were satisfactorily treated by solution nit. arg. of two scruples to the ounce of water, applied once a day by means of a nicely trimmed sponge upon a bent whalebone handle. The nostrils and throat must be kept open by the occasional use of sage tea, acidulated with vinegar.

*The post-mortem* examination of the body of Arthur Spring, executed for murder, was conducted, says the Philadelphia Medical and Surgical Journal, on June 11th, at the Philadelphia College of Medicine, by Prof. M'Clintock, and occupied almost two hours, being witnessed by a large audience of invited medical guests and students of the College. It was most interesting and satisfactory, as appearances were demonstrated that are by no means of usual occurrence under such circumstances, and an opportunity was afforded of showing the fallacy of the vulgar opinion as to these appearances. The criminal had died instantly, slight muscular tremors alone being observed, after the falling of the drop, and yet the universal idea that fracture must have occurred, with pressure upon the spinal chord, was in no wise correct. Neither was there cerebral congestion, common in victims of strangulation. The brain, ventricles and membranes, presented even less congestion and extravasation than is observed in a common febrile case. The conjunctiva was not reddened, the balls not protruded, lips not blackened, countenance not distorted; there was no effusion from mouth, ears or nostrils.

The skin, fasciæ and muscles of the neck were removed, and there blood was found in coagulated masses, lying between the muscles and around the vessels. The thyro-hyoid ligament was disruptured, and a large gap observed directly under the cord. The hyoid bone, epiglottis and tongue, were forced high up in the mouth. Every possibility of communication from the exterior, by the trachea into the lungs, was entirely prevented. The large vessels were disrupted and the respiratory nerves torn across. Here, then, was cause sufficient to account for the sudden death—instantaneous suffocation and suspension of innervation. The cervical vertebræ and ligaments were no ways unnatural; no disruption, discoloration or fracture had occurred, nor had the spinal marrow been at all infringed upon.

This case offers another powerful argument against the popular notion common even among professional men, that there must be cervical fracture. The suddenness of the death and the mobility of the head, induced many publicly to proclaim this as the cause of death, and nothing could have been happier than Dr. M'Clintock's demonstration of their error. In none of the instances of criminals executed in that county for the last five years, all of whom have been examined by Dr. M'C., has a fractured atlas or dentata ever been discovered, and in but one a dislocation from ruptured ligament.

Mammary Abscess.—Too little attention is paid, by the medical attendant, to these afflictions of the lying-in woman, in general practice. They are too often left to the care and conduct of the nurse, who knows nothing of the anatomy of the mammæ, or the arrangement of the lactiferous ducts. Too much care cannot be exercised to prevent this most painful affliction. The best prophylactic measures are to keep the breasts well exhausted, and elevated. This last should be particularly attended to when, from some cause, the mammæ become tumid and inflamed. Dr. Meigs has well said, that an inflamed breast needs a suspensory bandage as much as in orchitis. The method he suggests, and which we have used with success, is to suspend it with a strip of adhesive plaster wide enough to constitute a sling, and long enough to surround the breast, with the ends attached upon the region of the clavicle of that side. The strips should be passed under the breast, then this last raised gently upward, and then attach the ends. When the inflamed breast is allowed to hang, the lactiferous tubes above are diminished in their calibre by being stretched and elongated; while those below suffer a diminution by compression from the superincumbent weight.

---

